# Obesity & hypertension are determinants of poor hemodynamic control during total joint arthroplasty: a retrospective review

**DOI:** 10.1186/1471-2474-14-20

**Published:** 2013-01-14

**Authors:** Benedict U Nwachukwu, Jamie E Collins, Emily P Nelson, Mercedes Concepcion, Thomas S Thornhill, Jeffrey N Katz

**Affiliations:** 1 Harvard Medical School, 260 Longwood Avenue, Boston, MA, 02115, USA; 2 Orthopedic and Arthritis Center for Outcomes Research, Brigham and Women’s Hospital, 75 Francis St., Boston, MA, 02115, USA; 3 Department of Orthopedic Surgery, Brigham and Women’s Hospital, 75 Francis St., Boston, MA, 02115, USA; 4 Department of Anesthesia & Critical Care, Brigham and Women’s Hospital, 75 Francis St., Boston, MA, 02115, USA; 5 Division of Rheumatology, Immunology and Allergy, Brigham and Women’s Hospital, 75 Francis St., Boston, MA, 02115, USA; 6 Harvard School of Public Health, 677 Huntington Ave., Boston, MA, 02115, USA; 7 Department of Biostatistics, Boston University School of Public Health, 715 Albany Street, Talbot Building, Boston, MA, 02118, USA

**Keywords:** Hip, Knee, Blood pressure, Total joint arthroplasty, Obesity, Hypertension

## Abstract

**Background:**

Proper blood pressure control during surgical procedures such as total joint arthroplasty (TJA) is considered critical to good outcome. There is poor understanding of the pre-operative risk factors for poor intra-operative hemodynamic control. The purpose of this study is to identify risk factors for poor hemodynamic control during TJA.

**Methods:**

We performed a retrospective cohort analysis of 118 patients receiving TJA in the Dominican Republic. We collected patient demographic and comorbidity data. We developed an a priori definition for poor hemodynamic control: 1) Mean arterial pressure (MAP) <65% of preoperative MAP or 2) MAP >135% of preoperative MAP. We performed bivariate and multivariate analyses to identify risk factors for poor hemodynamic control during TJA.

**Results:**

Hypertension was relatively common in our study population (76 of 118 patients). Average preoperative mean arterial pressure was 109.0 (corresponding to an average SBP of 149 and DBP of 89). Forty-nine (41.5%) patients had intraoperative blood pressure readings consistent with poor hemodynamic control. Based on multi-variable analysis preoperative hypertension of any type (RR 2.9; 95% CI 1.3-6.3) and an increase in BMI (RR 1.2 per 5 unit increase; 95% CI 1.0-1.5) were significant risk factors for poor hemodynamic control.

**Conclusions:**

Preoperative hypertension and being overweight/obese increase the likelihood of poor blood pressure control during TJA. Hypertensive and/or obese patients warrant further attention and medical optimization prior to TJA. More work is required to elucidate the relationship between these risk factors and overall outcome.

## Background

Effective intraoperative control of blood pressure and heart rate during surgical procedures is considered crucial to good post-operative outcome. In fact, hemodynamic variability during cardiac procedures is associated with an increased incidence of adverse outcomes including perioperative mortality, stroke, myocardial infarction and other end-organ dysfunction [1-6]. Although there is less research on hemodynamic control during *non*-cardiac procedures, studies have suggested that increased intra-operative hemodynamic excursion (variations in heart rate and blood pressure from preoperative values) during non-cardiac procedures is similarly associated with adverse outcome [7,8]. Particularly with regard to orthopaedic surgery, Mortazavi et al. [9] suggest that intraoperative arrhythmia and blood pressure fluctuations are associated with increased risk of perioperative stroke after total joint arthroplasty (TJA).

Despite evidence that poor intra-operative hemodynamic control can lead to adverse consequences in non-cardiac surgeries, there is no consensus on management of perioperative hemodynamics [2]. *ACC/AHA 2007 Guidelines on Perioperative Cardiovascular Evaluation and Care for Noncardiac Surgery* state that for stage 3 hypertension, the potential benefits of delaying surgery to optimize blood pressure management with anti-hypertensives should be weighed against the risk of delaying the surgical procedure [10]. With no clearly established guideline there has been no attempt to identify risk factors leading to poor intra-operative hemodynamic control.

The goal of the present study was to investigate hemodynamic control during total hip and knee arthroplasty performed in a population with a high prevalence of hypertension preoperatively – patients receiving total joint arthroplasty in Santo Domingo, Dominican Republic. The patients were participants in Operation Walk Boston, a non-profit orthopedic mission trip that provides pro bono total joint replacement to Dominicans with advanced knee and hip arthritis. We sought to identify patient characteristics predictive of poor hemodynamic control. Patients with these risk factors should be monitored more closely and/or managed more intensively preoperatively to attenuate risk.

## Methods

### Study design overview

We performed a retrospective cohort study. With the approval of the Institutional Review Board (IRB) of Brigham and Women’s Hospital, we reviewed the anesthesia records of 118 patients receiving TJA as part of Operation Walk Boston between 2008 and 2011. Each mission trip was led by U.S. trained orthopaedic surgeons, anesthesiologists and auxiliary staff.

### Study population

Patients receiving surgery as part of the mission trips were selected in a multi-phase process. A Dominican orthopaedic surgeon initially identified patients with debilitating hip or knee arthritis who were not able to afford total joint replacement. This surgeon and a U.S. trained orthopaedic surgeon then examined this group of patients as well as their radiographs three months prior to the planned mission in order to confirm eligibility and identify the appropriate intervention. These patients subsequently underwent a preoperative evaluation in the Dominican Republic. These patients’ charts, including history and physical exam (performed by a doctor in the Dominican Republic), laboratory studies, EKG, and other relevant tests, were reviewed by a Boston-based team of anesthesiologists and orthopedic surgeons. Some patients were excluded at this point based on moderate to severe cardiac, endocrine, pulmonary, or renal pathology. Patients selected for surgery were seen again 1–5 days preoperatively as part of the mission team’s preoperative clinic. At the preoperative clinic, patients were evaluated by the mission trip’s nurses, physical therapists, anesthesiologists, internist and surgeons in order to be cleared for surgery. This preoperative evaluation included a focused history, physical examination and review of pertinent tests.

Patients were admitted from the preoperative clinic to the hospital to await surgery. Those presenting to the preoperative clinic with hypertensive urgency (defined for this mission as systolic blood pressures (SBP) >180 or diastolic blood pressures (DBP) >120) had intensification of their antihypertensive regimens in an attempt to achieve perioperative hemodynamic optimization. Patients received total knee replacement (TKR), total hip replacement (THR) or bilateral TKR or THR.

All THRs were uncemented. Tourniquets were used in all TKAs. During bilateral TKA cases, knees were operated on consecutively and thus tourniquets were never insufflated in both knees at the same time. We believe that the use of tourniquet has minimal effect on intra-operative blood pressure assessment in our study population.

### Data sources and data elements

As part of each preoperative clinic visit, a staff physician conducted a history and physical exam that was then recorded in preoperative clinic notes. We reviewed these clinic notes to abstract data on: patient demographics, body mass index (BMI), indication for procedure (including osteoarthritis, rheumatoid arthritis, avascular necrosis, congenital deformity and post-infectious), comorbidities (including hypertension and diabetes) and medications. Blood pressure and heart rate were also measured at these preoperative clinics. If the initial values were elevated, blood pressure and pulse were repeated after several minutes to account for effects of anxiety (“White Coat hypertension”). The blood pressure and heart rate measurements obtained at the preoperative visit served as our baseline value for each patient.

During each operative case, staff anesthesiologists completed a detailed, standardized anesthesia report that included 5-minute interval recordings of blood pressure and heart rate as well as perioperative and procedural parameters. We reviewed these anesthesia reports to abstract data on: American Society of Anesthesiologists (ASA) class, 5-minute interval hemodynamic recordings, presence of intra-operative rhythm disturbance, anesthesia type, timing of anesthesia induction, timing of incision and closure, use of intra-operative blood products, use of intra-operative vasopressors and use of intra-operative fluids.

We combined the data on history of hypertension and preoperative hypertension to create a three-level hypertension measure. Patients without a history of hypertension and without preoperative hypertension were classified as *no hypertension*. Patients with a history of hypertension and whose preoperative blood pressure was normal were classified as *controlled hypertension*. The remaining patients presented with hypertension preoperatively and were classified as *uncontrolled hypertension*. Some patients in the third group had history of hypertension and some did not. After review of bivariate and multivariable results we decided to collapse the first two groups to create a binary indicator: patients presenting with uncontrolled hypertension at the preoperative clinic versus those with normal preoperative blood pressures.

### Definition of hemodynamic control

Hypertension is defined as having systolic blood pressure equal to or greater than 140 mmHg and/or diastolic blood pressure equal to or greater than 90 mmHg. Our primary measure was mean arterial pressure (MAP), which combines systolic and diastolic blood pressures. MAP is equal to two-thirds diastolic blood pressure plus one-third systolic blood pressure. We retrospectively calculated MAP based on systolic and diastolic blood pressures abstracted from patient charts. For each 5-minute interval we defined poor hemodynamic control as a (MAP) falling above or below preoperative MAP by 35% (i.e. MAP <65% of preoperative MAP or >135% of preoperative MAP). Overall poor hemodynamic control for each patient was defined as 3 or more consecutive readings of poor hemodynamic control (i.e., MAP out of range for 15 or more minutes). There is no consensus in the literature on optimal intra-operative hemodynamic control but prior work in this area suggests that values falling outside of our pre-defined range significantly increases risk for peri/post-operative complication [11-13].

In addition to blood pressure and heart rate parameters we noted the frequency of intra-operative events signifying sub-optimal hemodynamic control: use of intraoperative blood products and intraoperative rhythm disturbance (atrial fibrillation, atrial flutter, premature contractions and abnormal rhythms). Intra-operative blood products refer to intra-operative transfusion of packed red blood cells. Utilization of intra-operative blood products signified sub-optimal hemodynamic control as these agents were utilized at the discretion of the anesthesiologist in cases where the patient had difficult hemodynamics and there was concern for inadequate tissue perfusion. Similarly, intraoperative rhythm disturbance was considered a proxy for sub-optimal hemodynamic control as we considered this to be potentially related to inadequate tissue perfusion.

### Statistical analyses

Study data were collected and managed using REDCap electronic data capture tools hosted by Partners HealthCare Research Computing, Enterprise Research Infrastructure & Services (ERIS) group. REDCap (Research Electronic Data Capture) is a secure, web-based application designed to support data capture for research studies [14]. Data were abstracted from records and recorded in a REDCap database and then exported to SAS for statistical analysis.

We used bivariate analysis to assess the unadjusted relative risk of poor hemodynamic control associated with several covariates. Covariates included: age, sex, BMI, history of hypertension, number of anti-hypertension medications, ASA class, indication for surgery, diabetes, anesthesia type and duration of surgery. We constructed a multivariable model to identify independent correlates of poor hemodynamic control. We used a modified Poisson regression with robust error variance to estimate the adjusted relative risk for each covariate considered. We used this approach rather than logistic regression, since the odds ratio can give a biased estimate for common outcomes [15]. We considered covariates that reached a relative risk ≥ 1.5 or ≤ 0.67 in bivariate analysis for inclusion into the multivariable model. Duration of surgery greater than 3 hours increased the risk for poor hemodynamic control (relative risk- 1.5), however given the strong and indistinguishable association between duration of surgery and procedure (bilateral vs unilateral) in our series, we did not include duration of surgery in multivariable models. All analyses were conducted using SAS software version 9.2 (SAS Institute, Cary NC).

### Funding sources

This study was funded through National Institutes of Health/National Institute of Arthritis and Musculoskeletal and Skin Diseases (NIH/NIAMS): NIH/NIAMS T32 AR 055885, P60 AR 47782.

## Results

### Descriptive data

We retrospectively reviewed intra-operative hemodynamic recordings for 118 patients undergoing TJA between 2008 and 2011. Average age at time of surgery was 60.1 years; 95 patients (81%) were female and 78 patients (74%) were overweight or obese (BMI ≥ 25). Average preoperative mean arterial pressure was 109.0 ± 13.4 mmHg [corresponding to an average SBP of 149 ± 22.1 mmHg and DBP of 89 ± 11.3 mmHg]. Overall, 20 patients had no history of hypertension and did not have hypertension preoperatively, 21 patients had a history of hypertension but did not have preoperative hypertension, and 76 patients presented to the preoperative clinic with hypertension. Of these 76 hypertensive patients, 55 patients had a prior diagnosis of hypertension and 21 had no prior history of hypertension (undiagnosed). There were 76 patients in total with a prior diagnosis of hypertension. Of those with a prior diagnosis of hypertension 39 patients were taking 2 or more anti-hypertensive medications and 6 were not taking any medications. Nine patients presented in a state of hypertensive urgency [SBP >180 or DBP >120] (Table 1). 

**Table 1:** Cohort characteristics

**Variable**	**Statistic**
Age group	
<55	29 (24.8%)
55-65	44 (37.6%)
>65	44 (37.6%)
Sex	
Male	23 (19.5%)
Female	95 (80.5%)
BMI group	
< 25	27 (25.7%)
25-30	40 (38.1%)
≥ 30	38 (36.2%)
Preop diastolic BP (mean (sd))	88.9 (11.3)
Preop systolic BP (mean (sd))	149.1 (22.1)
Preop mean arterial pressure (MAP) (mean (sd))	109.0 (13.4)
History of Hypertension	
No	42 (35.6%)
Yes	76 (64.4%)
Pre-operative Hypertension	
No	41 (35.0%)
Yes	76 (65.0%)
Hypertension group*	
None	20 (17.1%)
Controlled	21 (17.9%)
Uncontrolled	76 (65.0%)
Number of anti-Hypertensive meds (of those with a history of hypertension)	
0	6 (7.9%)
1	31 (40.8%)
2	23 (30.2%)
>2	16 (21.1%)
Hypertensive urgency**	
No	108 (92.3%)
Yes	9 (7.7%)
Diabetes	
No	100 (84.7%)
Yes	18 (15.3%)
ASA	
1	4 (3.4%)
2	86 (72.9%)
3	28 (23.7%)
Indication for Surgery***:	
Osteoarthritis	99 (83.9%)
Rheumatoid Arthritis	18 (15.3%)
Avascular Necrosis	3 (2.5%)
Congenital Deformity	2 (1.7%)
Other	5 (4.2%)
Procedure	
Unilateral TKR	52 (44.1%)
Unilateral THR	22 (18.6%)
Bilateral TKR	34 (28.8%)
Bilateral THR	9 (7.6%)
THR/TKR	1 (0.8%)
Anesthesia Type: Epidural	
No	107 (90.7%)
Yes	11 (9.3%)
Anesthesia Type: General	
No	115 (97.5%)
Yes	3 (2.5%)
Anesthesia Type: Spinal	
No	13 (11.0%)
Yes	105 (89.0%)
Duration of surgery group	
3 hours or less	98 (84.5%)
>3 hours	18 (15.5%)

* We combined the data on history of hypertension and preoperative hypertension to create a three-level hypertension measure. Patients without a history of hypertension and without preoperative hypertension were classified as *none*. Patients with a history of hypertension and whose preoperative blood pressure was normal were classified as *controlled*. The remaining patients with hypertension preoperatively were classified as *uncontrolled hypertension*. Thus some patients in the *uncontrolled* group had history of hypertension and some did not.

**SBP >180 or DBP >120.

***The sum of indications exceeds 100% because patients could have had more than one indication for surgery.

Forty-nine (41.5%) patients had intraoperative blood pressure readings consistent with poor hemodynamic control. Six patients (5.1%) required intra-operative blood products and 5 patients had an intra-operative rhythm disturbance (Table 2). 

**Table 2:** Outcomes

**Variable**	**Statistic**
Overall Poor hemodynamic control	
No	69 (58.5%)
Yes	49 (41.5%)
Intra-operative blood products	
No	112 (94.9%)
Yes	6 (5.1%)
Intra-operative rhythm disturbance	
No	113 (95.8%)
Yes	5 (4.2%)

### Bivariate analyses

Results of bivariate analysis (Table 3) suggest that those presenting to preoperative clinic with hypertension had an increased risk of poor hemodynamic control (RR 3.7; 95% CI 1.3 – 10.7) relative to patients without preoperative hypertension or a history of hypertension. Patients with controlled hypertension had a risk of poor hemodynamic control comparable to patients without any hypertension (RR 1.3; 95% CI 0.3 – 5.0). For this reason we collapsed the controlled and no hypertension groups and examined preoperative hypertension vs. no preoperative hypertension, disregarding history of hypertension. Those with preoperative hypertension had 3.2 times the risk of poor hemodynamic control relative to those without preoperative hypertension (95% CI: 1.6 – 6.5). 

**Table 3:** Results of Bivariate analysis

	**Poor hemodynamic control**		
**Variable**	**No**	**Yes**	**Relative Risk (95% CI)**
Age group			
<55	17 (58.6%)	12 (41.4%)	
55-65	29 (65.9%)	15 (34.1%)	0.8 (0.5, 1.5)
>65	22 (50.0%)	22 (50.0%)	1.2 (0.7, 2.0)
Sex			
Male	17 (73.9%)	6 (26.1%)	
Female	52 (54.7%)	43 (45.3%)	1.7 (0.8, 3.6)
BMI group			
<25	20 (74.1%)	7 (25.9%)	
25-30	23 (57.5%)	17 (42.5%)	1.6 (0.8, 3.4)
>30	20 (52.6%)	18 (47.4%)	1.8 (0.9, 3.8)
History of Hypertension			
No	29 (69.0%)	13 (31.0%)	
Yes	40 (52.6%)	36 (47.4%)	1.5 (0.9, 2.5)
Number of anti-Hypertensive meds			
0	34 (70.8%)	14 (29.2%)	
1	15 (48.4%)	16 (51.6%)	1.8 (1.0, 3.1)
2	12 (52.2%)	11 (47.8%)	1.6 (0.9, 3.0)
>2	8 (50.0%)	8 (50.0%)	1.7 (0.9, 3.3)
Pre-operative hypertension			
No	34 (82.9%)	7 (17.1%)	
Yes	34 (44.7%)	42 (55.3%)	3.2 (1.6, 6.5)
Hypertensive urgency			
No	66 (61.1%)	42 (38.9%)	
Yes	2 (22.2%)	7 (77.8%)	2.0 (1.3, 3.0)
Hypertension group			
None	17 (85.0%)	3 (15.0%)	
Controlled	17 (81.0%)	4 (19.0%)	1.3 (0.3, 5.0)
Uncontrolled	34 (44.7%)	42 (55.3%)	3.7 (1.3, 10.7)
ASA			
1	3 (75.0%)	1 (25.0%)	
2	50 (58.1%)	36 (41.9%)	1.7 (0.3, 9.3)
3	16 (57.1%)	12 (42.9%)	1.7 (0.3, 9.9)
Indication for Surgery: Rheumatoid Arthritis			
No	56 (56.0%)	44 (44.0%)	
Yes	13 (72.2%)	5 (27.8%)	0.6 (0.3, 1.4)
Diabetes			
No	60 (60.0%)	40 (40.0%)	
Yes	9 (50.0%)	9 (50.0%)	1.3 (0.7, 2.1)
Anesthesia Type: Spinal			
No	4 (30.8%)	9 (69.2%)	
Yes	65 (61.9%)	40 (38.1%)	0.6 (0.4, 0.9)
Duration of surgery group			
3 hours or less	61 (62.2%)	37 (37.8%)	
>3 hours	8 (44.4%)	10 (55.6%)	1.5 (0.9, 2.4)
Unilateral vs Bilateral			
Unilateral	46 (62.2%)	28 (37.8%)	
Bilateral	23 (52.3%)	21 (47.7%)	1.3 (0.8, 1.9)

Obese patients [BMI≥30] had a moderately increased risk of poor hemodynamic control relative to normal weight patients [BMI < 25] (RR 1.8; 95% CI 0.9-3.8). An increase of 5 BMI points conferred a significantly increased risk of poor hemodynamic control (RR 1.3; 95% CI 1.1-1.6). Patients with rheumatoid arthritis (RA) had a non-statistically significant decreased risk for poor hemodynamic control (RR 0.6; 95% CI 0.3 – 1.4).

Patients with duration of surgery greater than 3 hours were at increased risk for poor hemodynamic control (RR 1.5; 95% CI 0.9 – 2.4). The vast majority of cases over 3 hours were bilateral (17/18).

### Multivariable analysis

In the bivariate analysis, gender, BMI and preoperative hypertension reached the criterion for inclusion in a multivariable model (relative risk > 1.5 or < 0.67). Using multivariable analysis we found that preoperative hypertension of any type (RR 2.9; 95% CI 1.3-6.3) and an increase in BMI (RR 1.2 per 5 unit increase; 95% CI 1.0-1.5) were statistically significant risk factors for poor hemodynamic control. Women had a moderately increased risk of poor hemodynamic control compared to men (RR 1.7; 0.7 – 3.9), but this finding was not statistically significant.

## Discussion

In this study we sought to identify the risk factors for poor hemodynamic control during TJA. We found that preoperative hypertension and being overweight/obese were significant risk factors. The medical optimization of orthopaedic patients has received very little attention in the literature; however, our findings suggest that the hypertensive obese patient warrants further attention preoperatively.

Intra-operative hemodynamic control is considered an important driver of surgical outcome. Poor hemodynamic control has been reported in the cardiovascular literature to be associated with death, stroke, cognitive and renal dysfunction, perioperative myocardial infarction, and increased mortality [5,12,16,17]. Similarly, the non-cardiac literature also suggests that adequate intraoperative hemodynamic control is integral to outcome during non-cardiac procedures [6,7]. Physiologically, excess variability in intra-operative blood pressure can complicate intra-operative perfusion requirements and/or exacerbate bleeding risks. These data linking poor intra-operative hemodynamic control with adverse outcomes provide the justification for our use of poor hemodynamic control as the primary outcome variable in this study.

The risk for complication due to poor intra-operative blood pressure control during TJA is under-appreciated by the orthopaedic community. There is evidence in the orthopaedic literature that poor intra-operative blood pressure control is associated with increased risk for complication after TJA. One prior study found that systolic blood pressures above 150.6±9 mmHg significantly increased the risk of myocardial infarction in a cohort of patients undergoing a variety of non-cardiac procedures (orthopedic procedures represented greater than 50% of procedures in this study) [18]. Another study found that an intraoperative arrhythmia or other alteration in heart rate during TJA increased the risk of perioperative stroke [9]. To our knowledge no prior studies have systematically investigated risk factors for poor hemodynamic control during orthopedic procedures. Risk factors for poor blood pressure control identified in the primary care setting include: age, gender, obesity, race and presence of diabetes/impaired fasting glucose [19-21].

Our study highlights several key findings. First, we found that being overweight/obese was independently associated with an increased risk for poor hemodynamic control. The pathophysiologic cause of this association is unclear. Autonomic dysfunction in the obese patient could provide a plausible explanation [22]. Prior studies on surgical outcomes in overweight patients suggest that being overweight increases the risk of deep venous thrombosis as well as pulmonary embolus [23,24]. These prior observations in concert with the findings of the present study suggest that the overweight/obese patient undergoing orthopaedic surgery faces several distinct risks for adverse outcome. The present study finding is all the more pertinent given that the U.S. has the highest prevalence of obesity among developed nations (32% of the US adult population) [25]. In particular, given the association between obesity and OA, obese/overweight patients will likely represent a rapidly increasing segment of patients presenting for TJA. We note that obesity rates are increasing rapidly in developing countries [26] as well including the Dominican Republic where this study was conducted [27]. Managing the risks associated with obesity will be particularly challenging for the orthopaedic surgeon given the difficulty of perioperative weight loss in the osteoarthritic population.

Our study also demonstrates that preoperative hypertension is a major risk for poorly controlled hemodynamic control during total joint arthroplasty. This finding is congruent with prior work in this area. In particular, a landmark randomized multicenter study involving over 17,000 patients found that preoperative hypertension was associated with perioperative bradycardia, tachycardia and hypertension [28]. Despite evidence that pre-existing hypertension affects intra-operative blood pressure control there is no consensus on the impact of hypertension on perioperative outcome [29]. Some studies have suggested that perioperative hypertension has minimal impact on outcome while other studies have found that perioperative hypertension increases the risk for cardiac complication [18]. As such there is no consensus on management of the hypertensive patient prior to non-cardiac surgery [30]. Orthopaedic surgeons together with the anesthesiologist should adopt a strict approach for evaluating the orthopaedic surgery candidate. On the basis of our findings, we suggest a lower threshold of concern for the overweight/obese orthopaedic surgery patient presenting with hypertension. Ideally these patients should be medically optimized prior to surgery in order to attenuate risk and adverse outcome.

Our results suggest that duration of surgery increases the risk for poor hemodynamic control. However duration of surgery also appears to be strongly related to procedure performed and 17/18 surgeries greater than three hours in duration were bilateral in nature. Understanding the potential risks associated with bilateral and/or lengthy procedures is important and should be addressed in future investigations.

This study was set in the Dominican Republic, among patients with limited financial means who had a high prevalence of preoperative hypertension. Most of the patients with hypertension documented at the preoperative visit had history of hypertension but were not well controlled. Our data document the importance of attempting to optimize preoperative blood pressure control in patients such as the Operation Walk population who have a high burden of inadequately controlled hypertension. The data also indicate that mission trips such as Operation Walk Boston should coordinate with local physicians to ensure that patients selected for total joint arthroplasty are carefully optimized prior to admission for surgery. These efforts should include assessment of adherence to prescribed pharmaco-therapeutics as well as attention to non-pharmacologic approaches (dietary, lifestyle).

A potential limitation of the present study is that the small sample size precludes stable peri/postoperative outcome data such as deaths and cardiac and other complications. As such we do not report a complication rate. The literature suggests that poor hemodynamic control during surgery leads to adverse outcome; [1,7-9,11,18,29] however, without postoperative outcome data it is unclear whether this assumption holds true in our population. Another potential limitation of this study pertains to the preoperative measurement of hypertension. Blood pressure readings tend to be variable in nature and given the limited number of readings in the study population this introduces the possibility for misclassification. We are also aware that the findings in this entirely Dominican population may not be broadly generalizable. Finally, given the paucity of clinical data on intra-operative blood pressure control and its relationship to outcome, we cannot advocate our a priori definition of poor intra-operative blood pressure control as a standard definition.

This study focused on intra-operative blood pressure assessment during TJA. An area of further investigation is the perioperative blood pressure changes associated with TJA. In certain patient populations blood pressure control can be difficult immediately after the procedure and in the post-anesthesia care unit.

## Conclusions

Preoperative hypertension and being overweight/obese increase the likelihood of poor blood pressure control during TJA. Intraoperative blood pressure variability is an important determinant of perioperative outcome. We emphasize the need for careful preoperative assessment of the hypertensive and/or overweight/obese orthopaedic patient. We also highlight the importance of preoperative blood pressure monitoring and optimization for orthopedic mission programs performed in settings where hypertension is prevalent.

## Competing interests

The authors declare that they have no competing interests.

## Authors’ contributions

Conception: BUN; MC; TST JNK, Design: BUN; JEC; EPN; MC; TST; JNK, Data collection: BUN; EPN; MC, Data analysis: BUN; JEC; JNK, Manuscript draft & revision: BUN; JEC; EPN; MC; TST; JNK. All Authors have approved the final version of this manuscript in its entirety.

## Pre-publication history

The pre-publication history for this paper can be accessed here:

http://www.biomedcentral.com/1471-2474/14/20/prepub
